# Transcriptome Analysis Reveals the Potential Role of Long Noncoding RNAs in Regulating Fowl Adenovirus Serotype 4-Induced Apoptosis in Leghorn Male Hepatocellular Cells

**DOI:** 10.3390/v13081623

**Published:** 2021-08-17

**Authors:** Bo Wen, Xueping Wang, Lulu Yang, Ting Wang, Xiaolan Hou, Xuefeng Qi, Jingyu Wang

**Affiliations:** 1 College of Veterinary Medicine, Northwest A&F University, Xianyang 712100, China; bon@nwafu.edu.cn (B.W.); yangll@nwafu.edu.cn (L.Y.); t_wang@nwafu.edu.cn (T.W.); houxiaolan@nwafu.edu.cn (X.H.); 2 Anyang Institute of Technology, Anyang 455000, China; 20180051@ayit.edu.cn

**Keywords:** fowl adenovirus serotype 4, leghorn male hepatocellular cells, long noncoding RNA, RNA-seq, apoptosis

## Abstract

Hepatitis-hydropericardium syndrome (HHS) is caused by fowl adenovirus serotype 4 (FAdV-4) and has resulted in considerable economic losses to the poultry industry globally. FAdV-4 elicits apoptosis in host cells. Long noncoding RNAs (lncRNAs) have emerged as important regulatory RNAs with profound effects on various biological processes, including apoptosis. However, it remains unknown whether lncRNAs participate in FAdV-4-induced apoptosis. In this study, RNA sequencing was applied to determine the transcription of cellular lncRNA in leghorn male hepatocellular (LMH) cells infected with FAdV-4. Cellular RNA transcription analysis demonstrated that FAdV-4 infection elicited 1798 significantly differentially expressed (DE) lncRNAs in infected LMH cells at 24 h post-infection (hpi) compared to mock control infection. In addition, 2873 DE mRNAs were also found. Target prediction and analyses revealed that 775 DE lncRNAs whose 671 target mRNAs were among the DE mRNAs were involved in several signaling pathways, including the AMPK signaling pathway, p53 signaling pathway and insulin signaling pathway. From these 775 DE lncRNAs, we identified 71 DE lncRNAs related to apoptosis based on their target gene functions. Subsequently, lncRNA 54128 was selected from the 71 identified DE lncRNAs, and its role in FAdV-4-induced apoptosis was verified. LncRNA 54128 interference significantly suppressed the rate of apoptosis, which was accompanied by reduced BMP4 transcription levels. To the best of our knowledge, this is the first study to analyze host lncRNA transcription during FAdV-4 infection. Our findings provide a better understanding of host responses to FAdV-4 infection and provide new directions for understanding the potential association between lncRNAs and FAdV-4 pathogenesis.

## 1. Introduction

Fowl adenovirus serotype 4 (FAdV-4) is the primary causative agent of hepatitis–hydropericardium syndrome (HHS) [1]. Generally, 3–6-week-old broilers are especially vulnerable to HHS, resulting in a high mortality rate of 30–70% [2,3]. In addition, FAdV-4 has also been isolated from ducks [4], pigeons [5], quails [6] and geese [7] with clinical symptoms of HHS. The first outbreak of HHS was reported in Angara Goth, Pakistan in 1987, and subsequently, it has rapidly spread throughout the world to areas including Iraq [8], India [9], Japan [10], Mexico, Chile [11], Korea [1] and China [12,13]. In China, there were only sporadic outbreaks of HHS in poultry before 2015 [14]. Since then, however, the prevalence of HHS has been high with a dramatically increased number of typical clinical cases in broilers [15]. Due to the robust transmission and high pathogenicity of hypervirulent FAdV-4, HHS has resulted in considerable economic losses to the poultry industry [16], and the circumstances are still not optimistic [17]. Some advances have been achieved in diagnosis, prevention, isolation and identification as well as in the understanding of pathogenicity and epidemiology [14,18]. However, the understanding of the molecular mechanisms underlying the pathogenesis of FAdV-4 is superficial.

Apoptosis is a form of programmed cell death (PCD) in multicellular organisms and is tightly regulated by a multistep pathway. Apoptosis is characterized by cell shrinkage, chromatin condensation, nuclear fragmentation and cell fragmentation [19]. Viruses can escape the antiviral activity of the host and maintain their survival and replication by inducing apoptosis [20]. Therefore, apoptosis is closely associated with the pathogenesis of virus infection. It has previously been demonstrated that human adenoviruses can elicit apoptosis in host cells [21,22]. Recently, some reports have suggested that FAdV-4 infection leads to organ injury and dysfunction by activating the apoptotic machinery in infected cells [23,24]. PX, a structural protein of FAdV4, is an apoptosis inducer in leghorn male hepatocellular (LMH) cells [25].

Long noncoding RNAs (lncRNAs) are a set of RNAs longer than 200 nucleotides [26]. Due to the absence of a reading frame, lncRNAs do not have the ability to encode functional proteins, but can encode short polypeptides [27]. LncRNAs affect various physiological and pathological processes, such as differentiation [28], apoptosis [29], autophagy [30], development [31], tumorigenesis [32] and immune responses [33]. In recent years, accumulating evidence has indicated the involvement of lncRNAs in viral replication as positive or negative regulators through diverse mechanisms [34]. Moreover, viral infections also change the transcription profiles of lncRNAs in host cells to establish and maintain persistent infection [34,35,36,37]. In view of these findings, lncRNAs are an emerging hotspot in host–virus interaction research. Recently, several studies have investigated the roles of transcription products in the interaction between FAdV-4 and its host [38,39,40,41] by transcriptome analysis. However, the function of cellular lncRNAs during FAdV-4 infection is poorly understood.

In the present study, to explore the importance of lncRNA regulation in FAdV-4 infection-induced apoptosis, we performed next-generation sequencing to identify differentially expressed (DE) lncRNAs in LMH cells infected with FAdV-4. Our results indicated that FAdV-4 infection profoundly affected the transcription profile of lncRNAs in LMH cells. Bioinformatics analysis showed that some of the lncRNAs were likely involved in several signaling pathways, including the AMPK signaling pathway, p53 signaling pathway, insulin signaling pathway and pathways related to proteoglycans in cancer, revealing the vital functions of DE lncRNAs in FAdV-4-host interactions. Importantly, our data showed that lncRNA 54128 and its target gene BMP4 were co-expressed and that lncRNA interference 54128 significantly suppressed the rate of apoptosis, which was accompanied by a reduction in BMP4 transcription levels. In conclusion, this is the first study to demonstrate the transcription profiles and regulatory mechanisms of lncRNAs during FAdV-4 infection by next-generation sequencing methods. The data obtained in this study provide a valuable basis for further investigation of the roles of lncRNAs in FAdV-4 infection and pathogenesis.

## 2. Materials and Methods

### 2.1. Cells, Viruses and Antibodies

The LMH cell line was kindly gifted by Prof. Yunfeng Wang (Harbin Veterinary Research Institute, Heilongjiang, China). Cells were cultured in Dulbecco’s Minimal Essential Medium (Gibco, New York, NY, USA) containing 10% fetal bovine serum (Gibco, Grand Island, NE, USA), 100 IU/mL penicillin and 10 μg/mL streptomycin (HyClone USA) at 37 °C in an incubator with 5% CO_2_. Cell culture plates were precoated with 2 mL of a 0.1% gelatin solution (Millipore, Billerica, MA, USA) and incubated at 4 °C for 20 to 30 min before they were used to culture cells. FAdV-4 SX17 (GenBank: MF592716.1) was isolated and stored by our laboratory. Virus was amplified by collecting the infected cell supernatant when approximately 80% of the cells showed a cytopathic effect (CPE). Cells were freeze–thawed three times and stored in aliquots at −80 °C. According to the method of Reed and Muench, the titers of the harvested virus were determined and expressed as the 50% tissue culture infective dose (TCID_50_)/mL. A rabbit polyclonal anti-FAdV-4-hexon antibody was generated by our laboratory. The anti-β-actin (HC201) primary antibody was purchased from TransGen Biotech (Beijing, China). Horseradish peroxidase-conjugated goat anti-mouse (A9917) and anti-rabbit (A0545) secondary antibodies were purchased from Sigma-Aldrich (St. Louis, MO, USA). Anti-caspase-3 (19677-1-AP) and anti-PARP1 (13371-1-AP) antibodies were purchased from Proteintech (Chicago, IL, USA).

### 2.2. Virus Infection

LMH cells were cultured in 12- or 6-well plates and allowed to grow for approximately 24 h to reach approximately 80% confluence. Cells were then washed three times with phosphate-buffered saline (PBS) and infected with the FAdV-4 isolated strain at the indicated multiplicity of infection (MOI). LMH cells inoculated with similarly purified and triple freeze–thaw prepared LMH cells were used as the mock-infected group. After 1 h of incubation at 37 °C, unbound viruses were removed by washing three times with PBS, and the infected cells were maintained in 2% FBS DMEM. Samples were harvested at the indicated time points for further experiments. Three independent biological replicates of the FAdV-4- and mock-inoculated groups were prepared at each time point for all experiments.

### 2.3. Immunoblot Analysis

LMH cells were infected with FAdV-4 SX17. Protein samples prepared from harvested cells were subjected to immunoblotting using primary antibodies. After adding 5× SDS-PAGE sample buffer to cell lysates, the samples were boiled for 10 min, separated by SDS-PAGE and then transferred onto 0.22-µm polyvinylidene difluoride membranes (Millipore, Billerica, MA). The membranes were blocked with 5–10% nonfat milk and incubated with the indicated primary antibodies followed by HRP-conjugated secondary antibodies. The bound antibodies were detected with enhanced chemiluminescence (ECL) immunoblotting detection reagents (Millipore, Billerica, MA, USA), and images were obtained with a CanoScan LiDE 100 scanner (Canon).

### 2.4. Transfection of siRNAs and Gene Silencing

The siRNA for lncRNA 54128 was synthesized by Ribo Biotechnology. LMH cells grown to 80% confluence in 12-well cell culture plates were transfected with 50 nM siRNA using TurboFect Transfection Reagent (Thermo Fisher Scientific, Waltham, MA, USA) according to the manufacturer’s instructions. Cells were cultured in 5% CO_2_ at 37 °C for 24 h. The reaction mixture was discarded, and cells were then infected with FAdV-4 at a MOI of 0.1. Following 1 h of incubation with FAdV-4, cells were incubated in fresh medium until collection at the indicated time points after infection. The silencing efficiency was measured with qRT-PCR.

### 2.5. TUNEL Staining

Apoptotic events postinfection were examined by one-step terminal deoxynucleotidyl transferase-mediated dUTP biotin nick end labeling (TUNEL) assays. At the indicated postinfection times, cells were washed four times with PBS and fixed in 4% paraformaldehyde. The staining procedures were performed according to the manufacturer’s instructions (Beyotime Institute of Biotechnology, Haimen, China), and cells were analyzed under a confocal microscope (CLSM Leica SP8, Wetzlar, Germany). The number of positive cells was counted in three randomly selected fields of view using three sections from each sample.

### 2.6. Flow Cytometry Analysis

After lncRNA 54128 was knocked down in infected LMH cells, flow cytometry was employed to determine apoptosis using an annexin V−FITC double-staining apoptosis detection kit (Beyotime Institute of Biotechnology, Haimen, China) according to the manufacturer’s protocol. After the knockdown cells were infected with FAdV-4 at a MOI of 0.1, they were collected at 36 h postinfection (hpi) and then washed three times with PBS. Subsequently, washed cells were centrifuged, suspended in 500 μL of 10× binding buffer and treated with 10 μL of FITC-labeled annexin V per sample for 10 min at room temperature. The infected cells were then stained with 5 μL of propidium iodide (PI) per sample for 5 min. Finally, a Coulter Epics XL FACS system (Beckman Coulter, Brea, CA, USA) was utilized to evaluate the treated cells. Annexin V-positive and PI-negative cell populations in the lower right quadrant of the annexin V versus PI FACS plots were considered apoptotic cells.

### 2.7. Library Construction and Quality Control

After infection with FAdV-4 at a MOI of 0.1, LMH cells were harvested at 24 hpi. Subsequently, total RNA was extracted from 6 samples (three FAdV-infected and three mock-infected samples) using TRIzol reagent (Invitrogen, Carlsbad, CA, USA) according to the manufacturer’s protocol. A NanoPhotometer spectrophotometer (NanoDrop products IMPLEN, CA, USA) was used to measure the quantity and purity of the total RNA, and RNA integrity was tested by the Bioanalyzer 2100 system (Agilent Technologies, CA, USA). mRNAs and lncRNAs were then enriched by removing ribosomal RNAs (rRNAs) from qualified total extracted RNA with the Ribo-Zero Magnetic kit (EpiCentre). Enriched mRNAs and lncRNAs were fragmented into short fragments. From these short RNA fragments, first-strand cDNA was synthesized with hexamer random primers, and second-strand cDNA was generated by substituting dTTP with dUTP. The cDNA fragments were then purified and ligated to adapters, and the second-strand cDNA was digested using uracil-N-glycosylase (UNG). After agarose gel electrophoresis, suitable fragments were selected as templates for PCR amplification. The final cDNA library quality was assessed on a Qubit 2.0 Fluorometer (Life Technologies, Carlsbad, CA, USA) and the Agilent Bioanalyzer 2100 system (Agilent Technologies, Santa Clara, CA, USA). Finally, 6 high-quality libraries were obtained and sequenced on an Illumina HiSeq 2500 (Illumina, San Diego, CA, USA).

### 2.8. RNA Sequencing and Data Processing

After RNA sequencing, raw reads from each library were produced and then filtered by removing adapter reads, low-quality reads and reads containing over 10% ambiguous residues (Ns) to obtain clean reads. Next, the Q20, Q30 and GC contents were monitored to evaluate the clean reads. Subsequently, the clean reads were aligned and mapped to the chicken reference genome (genome assembly: Gallus_gallus-5.0 GCA_000002315.3) using Hisat2 (version 2.0.4) under a spliced mapping algorithm with default parameters [42]. Cufflinks (V2.2.1) [43] was used to reconstruct transcripts and generate the final comprehensive set of transcripts with the mapped reads. To detect novel transcripts, all assembled transcripts were aligned with reference annotation utilizing Cuffcompare [43]. The novel transcripts met the following parameters: (1) transcript length longer than 200 bp and (2) exon number more than 2. To identify novel lncRNA transcripts, protein-coding transcripts, microRNAs (miRNAs), tRNAs, snoRNAs, and rRNAs were first filtered according to genome annotation information. The following criteria were used to predict novel lncRNAs: (1) exon number ≥ 2, (2) length > 200 nucleotides, (3) FPKM ≥ 0.5, (4) lack of coding capacity, and (5) no overlap with mRNAs or annotated lncRNAs. Coding ability was predicted using the Coding–Non-Coding Index (CNCI), Coding Potential Calculator (CPC) and Coding Potential Assessment Tool (CPAT). For gene transcription analysis, matched reads were calculated and then normalized to RPKM values using RSEM (Li and Dewey, 2011). Gene FPKMs were calculated by summing the FPKMs of transcripts in each gene group. Differential transcription analysis of two groups was performed using the DESeq R package (1.8.3). *p*-value < 0.05 and |log2(fold change)| ≥ 1 were set as the thresholds for significantly differential transcription by default.

### 2.9. Prediction of DE lncRNA Target Genes

To predict the target genes of DE lncRNAs, analyses of cis and trans regulation were performed. A cis-acting role is one in which the lncRNA acts on neighboring target genes. Prediction of a cis-acting target gene suggests that the function of the lncRNA is related to the protein-coding genes adjacent to the coordinate. To identify cis regulation, the genes located within a 10-kb window upstream or downstream of the lncRNAs were classified as cis target genes. In addition, the target mRNAs operating under trans regulation were identified by the RNAplex software [44].

### 2.10. GO and KEGG Pathway Analyses

Gene ontology (GO) and Kyoto Encyclopedia of Genes and Genomes (KEGG) analyses were performed to identify biological processes and pathways associated with the cis and trans target genes of the DE lncRNAs. A false discovery rate (FDR) was used to correct the *p*-values. A corrected *p*-value (Q value) < 0.05 was considered significant.

### 2.11. qRT-PCR

Relative quantification methods were employed to validate the transcription of DE mRNAs and DE lncRNAs. The primers are listed in Table 1. According to the manufacturer’s instructions, TRIzol reagent was used to extract the total RNA of LMH cells, and reverse transcription was then performed using M-MLV reverse transcriptase (TransGen Biotech, Beijing, China). qRT-PCR was performed using SYBR Green master mix (TransGen Biotech, Beijing, China) on an iQ5 qRT-PCR System (Bio-Rad, Hercules, CA, USA). The PCR cycling conditions were 2 min at 95 °C followed by 40 cycles of 15 s at 94 °C and 45 s at 60 °C. The differences between the target gene and reference gene were calculated using the 2^−ΔΔCt^ method. The relative transcription level of each gene was normalized to that of GAPDH.

### 2.12. Statistical Analysis

All values are expressed as the arithmetic mean of triplicates ± standard error of the mean (SEM). Significance was determined by one-way ANOVA with a Dunnett posttest or by paired Student’s *t*-test. Values of *p* < 0.05 were considered to indicate statistical significance.

## 3. Results

### 3.1. FAdV-4 Replicates and Induces Apoptosis in LMH Cells

**Figure 1:**
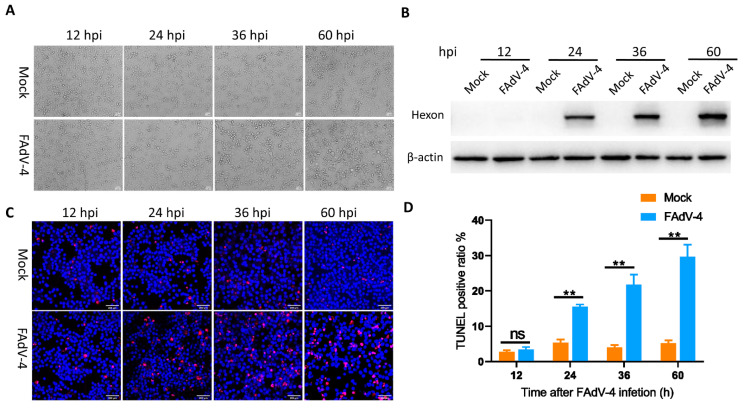
FAdV-4 replicated and induced apoptosis in LMH Cells. (**A**) Morphological changes in LMH cells at different time points after FAdV-4 infection (MOI = 0.1), with mock-infected cells as a control. (**B**) Western blot analysis of hexon levels in FAdV-4-infected LMH cells at indicated time points post inoculation. (**C**) TUNEL labeling of FAdV-4-infected LMH cells. LMH cells were mock-infected or infected with FAdV-4 (MOI = 0.1) for 12, 24, 36 and 60 h. The cell samples were labelled with TUNEL (red) and then counterstained with Hoechst 33342(blue) solution. Scale bars, 100 μm. (**D**) Percentage of TUNEL-positive mock-infected and FAdV-4-infected LMH cells at the indicated time points. The data represent the mean ± SD of three independent experiments. *P* values were calculated using Student’s *t*-test. An asterisk indicates a comparison with the indicated control. ** *p* < 0.01, n.s., not significant.

To determine the kinetics of FAdV-4 replication in host cells, we first infected LMH cells with FAdV-4 at a MOI of 0.1 and then detected CPEs at 12, 24, 36 and 60 hpi. The expression levels of viral hexon protein were detected by immunoblotting assays at 12, 24, 36 and 60 hpi. Compared to mock-infected cells, FAdV-4-infected cells exhibited ballooning and clumping at 24 hpi. From 36 to 60 hpi, almost all FAdV-4-infected cells were swollen and round-shaped (Figure 1A) compared to mock-infected cells. In addition, hexon protein in FAdV-4-infected cells was detected as early as 24 hpi, and its expression showed an increase from 24 to 60 hpi (Figure 1B). Additionally, TUNEL labeling indicated that the apoptotic cell ratio of virus-infected cells began to increase at 24 hpi, and the percentage of apoptotic cells increased with infection time, reaching a maximum of 29.7% at 60 hpi (Figure 1C,D) compared to mock-infected cells.

FAdV-4 induces apoptosis of infected cells [23,24,25], and many host factors regulate the process of apoptosis, including lncRNAs. However, it remains unknown whether lncRNAs participate in FAdV-4-induced apoptosis. Therefore, we utilized high-throughput sequencing to identify host lncRNAs implicated in regulating apoptosis. In the present study, high expression levels of hexon protein indicated high virus levels in infected cells at 24 hpi. FAdV-4-infected cells had a significantly higher level of apoptosis than mock-infected cells as early as 24 hpi. As the interaction between the adenovirus and host has been reported to mainly occur before the rapid proliferation of the virus [45], FAdV-4- and mock-infected cells were harvested at 24 hpi in triplicate for library construction and lncRNA sequencing.

### 3.2. Overview of RNA Sequencing Data

To investigate lncRNA profile changes in LMH cells infected with FAdV-4, 6 strand-specific libraries were successfully constructed, and high-throughput sequencing obtained 621,217,628 raw reads from all samples. After removing adapter reads, low-quality reads and reads matching rRNA, 619,305,456 clean reads remained in total with Q30 values ranging from 92.28% to 94.25% (Table 2). Subsequently, the clean reads were mapped to the chicken genome, resulting in mapping ratios from 73.189% to 92.545% for all samples (Table 2).

### 3.3. Differential Transcription Analysis of lncRNAs and mRNAs

**Figure 2:**
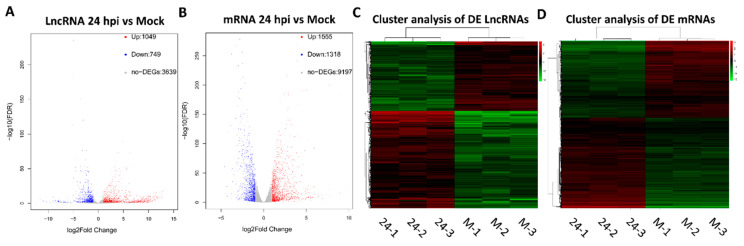
Differential transcription of lncRNAs and mRNAs in FAdV-4-infected and mock-infected LMH cells. Volcano plot diagram of the DE lncRNAs (**A**) and mRNAs (**B**) between FAdV-4 and mock infected cells. Hierarchical clustering of the DE lncRNAs (**C**) and mRNAs (**D**).

To identify significant DE lncRNAs or mRNAs between the mock- and FAdV-4-infected cells, a *p* value < 0.01 and a |log2 (fold change)| > 1 were used as the cutoff values. As a result, 1049 lncRNAs and 1555 mRNAs were upregulated, while 749 lncRNAs and 1318 mRNAs were downregulated in the FAdV-4-infected cells compared to the mock-infected cells (Figure 2A,B). Hierarchical clustering indicated that the transcription levels of DE lncRNAs and mRNAs in infected cells were significantly different than those in mock-infected cells, showing that the differential transcription of these lncRNAs and mRNAs was caused by viral infection (Figure 2C,D).

### 3.4. GO and KEGG Pathway Analyses of DE mRNAs

**Figure 3:**
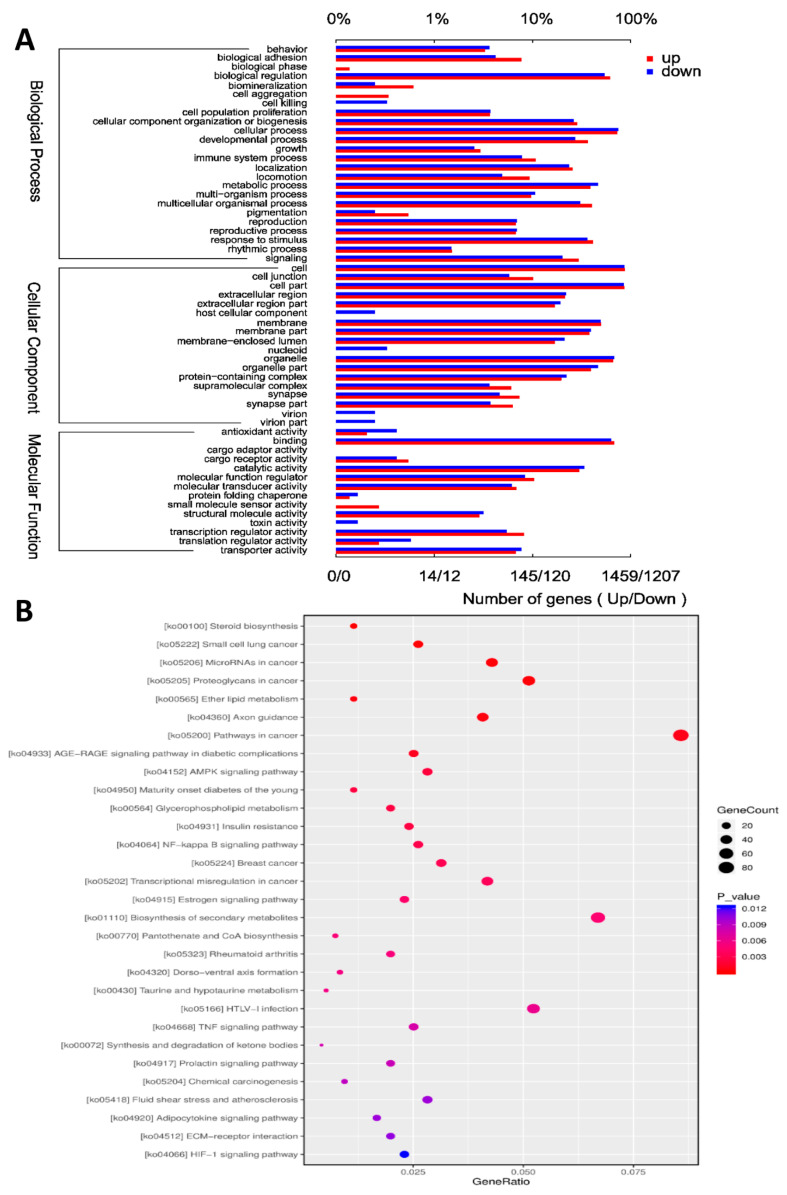
GO and KEGG pathway analyses of DEmRNAs. (**A**) GO analysis of the DE mRNAs. The *X* axis shows the number of DE genes, and the *Y* axis shows the GO terms. All GO terms are grouped into biological process, cellular components and molecular function. The red bar represents the number of up-regulated genes, and the blue bar represents the number of down-regulated genes. (**B**) KEGG analysis of the DE mRNAs. In this graphic, the degree of KEGG enrichment is assessed by the GeneRatio, *p*-value, and GeneCount. The closer the *p*-value is to zero, the greater the GeneRatio is. The greater the GeneCount is, the more significant the enrichment is.

To better understand the molecular functions and biological processes of the DE mRNAs in FAdV-4-infected cells, GO annotation was performed to elucidate their biological functions. According to the GO annotation, the 2873 annotated DEGs showed enrichment of 56 GO terms, including 24 biological process (BP), 18 cellular component (CC) and 14 molecular function (MF) GO terms (Figure 3A). To further analyze the roles that these DE mRNAs might play in regulatory networks, we also assessed their enriched KEGG pathways. As a result, 344 KEGG pathways were identified, including the NF-κB signaling pathway, apoptosis pathway, TNF signaling pathway, AMPK signaling pathway and IL-17 signaling pathway. The top 30 enriched pathways of the DE mRNAs are presented in Figure 3B. These results indicated that the DE mRNAs may play a crucial role in virus−host interactions.

### 3.5. Functional Prediction of the DE lncRNAs and Identification of lncRNAs Related to Apoptosis

**Figure 4:**
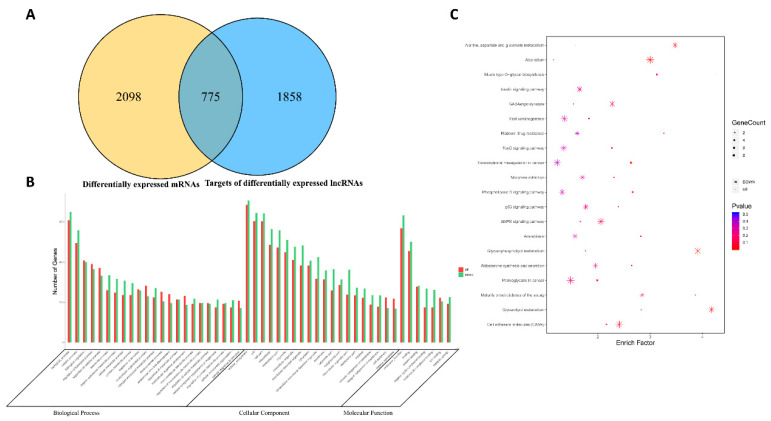
Functional prediction of DE LncRNAs and identification of LncRNAs related to apoptosis. (**A**) Venn diagram shows the number of overlap genes in target genes of differentially expressed lncRNAs. (**B**) GO analysis of the overlap genes in target genes of differentially expressed lncRNAs. The *Y* axis shows the number of DE genes, and the *X* axis shows the GO terms. All GO terms are grouped into biological process, cellular components and molecular function. The red bar represents the number of up-regulated genes, and the blue bar represents the number of down-regulated genes. (**C**) KEGG analysis of the overlap genes in target genes of differentially expressed lncRNAs. In this graphic, the degree of KEGG enrichment is assessed by the GeneRatio, *p*-value, and GeneCount. The closer the *p*-value is to zero, the greater the GeneRatio is. The greater the GeneCount is, the more significant the enrichment is.

To some extent, the function of lncRNAs can be inferred through their associated cis-regulated and trans-regulated mRNAs [46]. Therefore, we not only searched for protein-coding genes within 100 kb of each DE lncRNA as cis target genes, but also used RNAplex software to predict target mRNAs as trans target genes. As a result, 2633 target genes for 595 known lncRNAs and 734 novel lncRNAs with differential transcription between the mock-infected and FAdV-4-infected groups were predicted. We subsequently used the overlapping DE lncRNA target genes and DE mRNAs for further investigation, and we found that 775 DE mRNAs (Figure 4A) were targeted by 671 DE lncRNAs. We employed GO and KEGG analyses of the 775 DE mRNAs to further annotate their functions. According to the results of the GO analysis, the target genes of the selected DE lncRNAs were mainly associated with cellular process, biological process, cell parts, protein binding and catalytic activity (Figure 4B). In the KEGG pathway analysis, most of the target genes of the selected DE lncRNAs were involved in the AMPK signaling pathway, p53 signaling pathway, insulin signaling pathway and pathways related to proteoglycans in cancer (Figure 4C). To further explore the role of lncRNAs in FAdV-4 infection-induced apoptosis, 71 DE lncRNAs related to apoptosis were acquired according to the functions of the target genes of the selected DE mRNAs (Table S1). These lncRNAs were further screened using the following conditions: fold change greater than 1.5 for lncRNAs related to apoptosis and lncRNA target genes; and Pearson correlation greater than 0.90 for lncRNAs and mRNAs. After screening, we obtained 20 lncRNAs, which are listed with their target mRNAs in Table 3.

### 3.6. Validation of the lncRNA−mRNA Pairs by qRT-PCR

To validate the predicted lncRNA−mRNA pairs related to apoptosis, we randomly selected five DE lncRNAs and their four target mRNAs (Table 2) for qRT-PCR analysis. As a result, the relative transcription levels of the selected lncRNAs and their target mRNAs detected by qRT-PCR showed trends similar to those of RNA sequencing (Table 4), indicating that the data obtained from sequencing and the prediction of lncRNA−mRNA pairs were relatively reliable and accurate.

### 3.7. LncRNA 54128 and BMP4 Are Upregulated during the Course of FAdV-4 Infection

**Figure 5:**
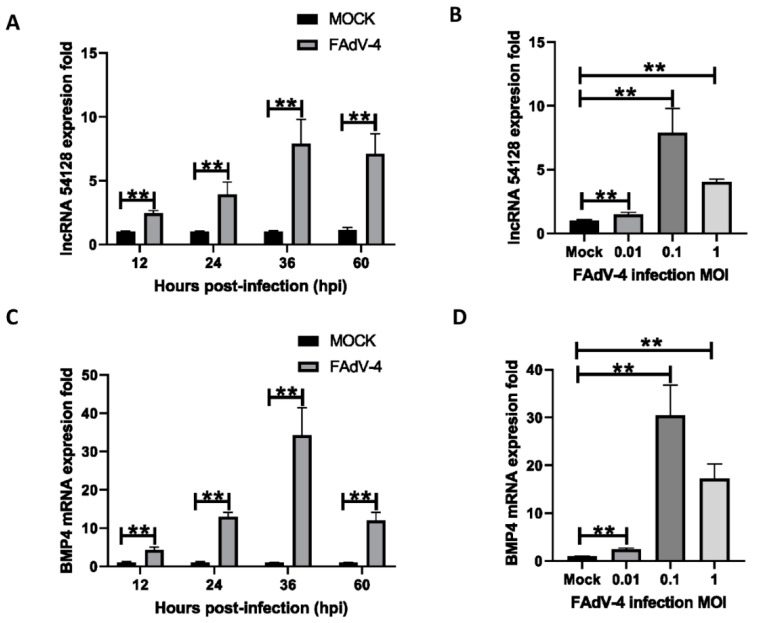
LncRNA 54218 and BMP4 upregulation over the course of FAdV-4 infection. (**A**,**C**) LMH cells were infected with FAdV-4 (MOI = 0.1) for 12, 24, 36 and 60 h, and the transcription levels of LncRNA 54218 and BMP4 were determined by qPCR. (**B**,**D**) LMH cells were infected with FAdV-4 (MOI = 0.01, 0.1, 1) for 36 h, and the transcription levels of LncRNA 54218 and BMP4 were determined by qPCR. The data represent the mean ± SD of three independent experiments. *p* values were calculated using Student’s *t* test. An asterisk indicates a comparison with the indicated control. ** *p* < 0.01, n.s., not significant.

To study the role that lncRNAs play in FAdV-4-induced apoptosis, lncRNA 54128 was selected for further study from the 71 identified lncRNAs whose functions were predicted to be involved in apoptosis. We first explored the correlation between FAdV-4 infection and lncRNA 54128 by examining the lncRNA 54128 transcription levels in LMH cells infected with FAdV-4. LncRNA 54128 was upregulated in a virus dose- and infection time-dependent manner. At a MOI of 0.1, significant upregulation was observed 12 h after infection and peaked at 36 h (Figure 5A). At 36 h after infection at a MOI of 0.1, the lncRNA 54218 transcription levels peaked (Figure 5B). Interestingly, BMP4, a lncRNA 54128 target mRNA, was also upregulated in a virus dose-dependent manner (Figure 5C) and in an infection time-dependent manner (Figure 5D), showing the same transcription trends as lncRNA 54128.

### 3.8. Effect of Silencing lncRNA 54128 on FAdV-4-Induced Apoptosis

**Figure 6:**
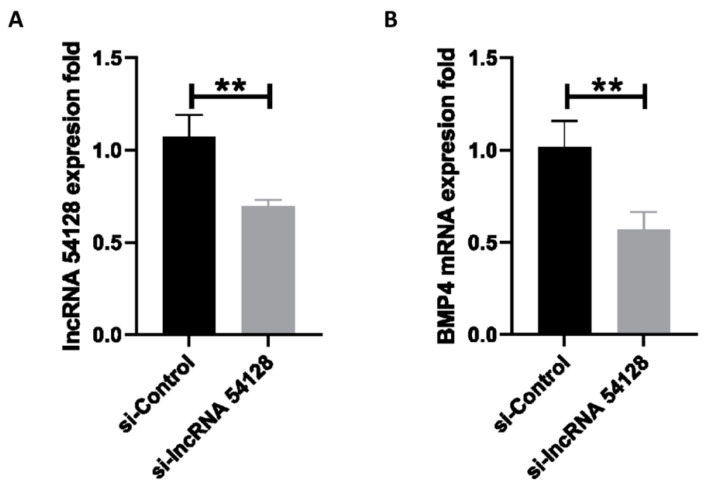
Effect of silencing LncRNA 54218 on BMP4 transcription. LMH cells were transfected with either the siControl or si LncRNA 54218 and then infected with FAdV-4 (MOI = 0.1) for 36 h. The transcription levels of (**A**) LncRNA 54218 and (**B**) BMP4 were analyzed by RT-qPCR. The data represent the mean ± SD of three independent experiments. *p* values were calculated using Student’s *t* test. An asterisk indicates a comparison with the indicated control. ** *p* < 0.01, n.s., not significant.

**Figure 7:**
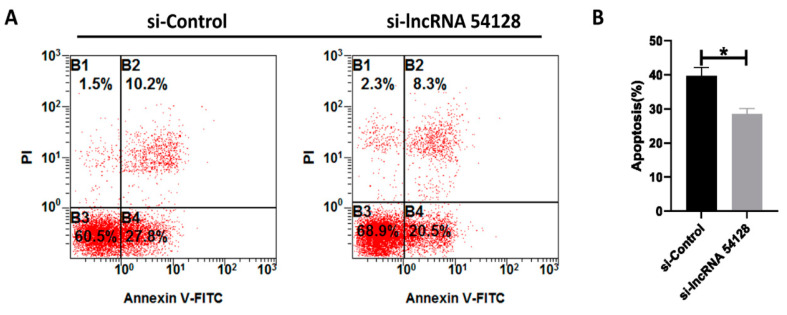
Downregulation of LncRNA 54218 specifically inhibits apoptosis in LMH cells with FAdV-4 infection. LMH cells were transfected with either the siControl or si LncRNA 54218 and then infected with FAdV-4 (MOI = 0.1) for 36 h. (**A**) The cell samples were dual-labelled with Annexin V and PI and analyzed by flow cytometry. (**B**) The proportions of apoptotic cells among the total cells. The data represent the mean ± SD of three independent experiments. *p* values were calculated using Student’s *t* test. An asterisk indicates a comparison with the indicated control. * *p* < 0.05; n.s., not significant.

To illustrate the association of lncRNA 54128 with its target gene, lncRNA 54128 was knocked down in LMH cells using a specific siRNA. The siRNA for lncRNA 54128 was effective in reducing the transcription of the lncRNA compared to the negative control (Figure 6A). Moreover, lncRNA 54128 interference significantly suppressed the transcription of BMP4 (Figure 6B). To determine whether the reduction in lncRNA 54128 transcription plays a role in FAdV-4 infection-induced apoptosis, the rates of apoptosis in LMH cells after infection were analyzed by flow cytometry (Figure 7A). Surprisingly, silencing lncRNA 54128 decreased the apoptotic rates of infected LMH cells compared to control siRNA cells (Figure 7B). Therefore, these results suggested that lncRNA 54128 plays a positive role in FAdV-4 infection-induced apoptosis by regulating the transcription of BMP4, a target gene of lncRNA 54128.

## 4. Discussion

FAdV-4, a hepatotrophic virus, is the causative agent for HHS in chickens. Since 2015, the prevalence of severe HHS has increased in China and has resulted in considerable economic losses in the poultry industry [13,47]. However, the underlying molecular mechanisms of FAdV-4 pathogenesis are still poorly understood. As a powerful research tool, transcriptome analysis has been used widely to reveal the interaction between host and virus. Based on this technology, several studies have revealed that changes in cell gene transcription are an important aspect of the interaction between FAdV-4 and its host [38,40,41]. It is worth noting that our previous study showed that the binding and entry of FAdV-4 into LMH cells profoundly affect early cellular miRNA transcription profiles and that cellular miRNAs play an important role in FAdV-4 entry [39]. As another type of noncoding RNA, lncRNAs play an important regulatory role in the battle between virus and host, involving the transcription of viral genes, transcription of host genes, stability of mRNAs, translation of mRNAs and host antiviral response [48]. Although the key role of individual lncRNAs in pathogenesis is increasingly being recognized, to our knowledge, the lncRNA transcription profile in host LMH cells affected by FAdV-4 infection and the possible role of lncRNAs in FAdV-4−host interactions have not been investigated. In this study, we analyzed the dynamics of lncRNA transcription during viral infection. Our results identified a total of 1798 DE lncRNAs between the FAdV-4-infected group and the mock-infected group, suggesting a specific host response to FAdV-4 infection. To the best of our knowledge, this is the first transcriptome analysis of host lncRNA and mRNA variations in FAdV-4 infection.

In the present study, the overlap of DE lncRNA target genes and DE mRNAs acquired from transcriptome analysis was utilized to predict the biological function of DE lncRNAs, resulting in relatively more reliable target relationships between lncRNAs and mRNAs. Our results indicated that the function of the DE lncRNAs in FAdV-4-infected cells was predicted to involve the regulation of the FOXO signaling pathway, p53 signaling pathway and AMPK signaling pathway. It has been reported that these signaling pathways may play important roles in regulating viral infection [49,50]. p53 regulates various cellular responses, including apoptosis, cell cycle arrest, senescence, DNA repair, metabolism, antioxidant defense, autophagy and ferroptosis, and all of these roles contribute to the role of p53 in tumor suppression [51,52,53]. p53-dependent cell death/apoptosis is required for productive adenovirus infection [54]. Additionally, the AMPK signaling pathway coordinates cell growth, autophagy and metabolism [55]. Human adenovirus 36 infection substantially increases Cidec/FSP27, but significantly reduces AMPK activity in primary cultured human skeletal muscle cells [56]. All these functional analyses suggest that cellular lncRNAs have profound effects on the regulation of FAdV-4 infection by regulating target genes.

Infection with pathogenic viruses may activate the apoptotic machinery in infected cells [21]. It has been reported that FAdV-4 infection induces apoptosis in the liver and apoptosis of LMH cells in vitro [57]. PX, a structural protein of FAdV-4, is an apoptosis inducer in LMH cells [57]. In our study, we also demonstrated that FAdV-4 infection of LMH cells induced apoptosis. Although it has been reported that lncRNAs regulate apoptosis [29], the roles of lncRNAs during FAdV-4-induced apoptosis have not yet been elucidated. In this study, we identified 65 DE mRNAs related to apoptosis. We further found that these mRNAs were targeted by 71 DE lncRNAs, indicating that these 71 DE lncRNAs are apoptosis-related lncRNAs. Among the targeted relationships, BMP4 was found to be targeted by ENSGALG00000055015, XLOC_033831 and ENSGALG00000054128. MKP3 was found to be targeted by XLOC_003734 and XLOC_000612. FOXO3 was found to be targeted by XLOC_026155 and ENSGALG00000050472. It has been reported that restoring BMP4 expression in vascular endothelial progenitors ameliorates maternal diabetes-induced apoptosis and neural tube defects [58], and BMP4 directly induces caspase-3-mediated apoptosis in neurons and oligodendrocytes in vitro. Additionally, the downregulation of cytosolic MKP-3 maintains ERK1/2 activity and inhibits the execution of apoptosis by preventing Bcl-2 degradation and mitochondrial release of cytochrome [59]. Acetylshikonin induces apoptosis in HCT-15 and LoVo human colorectal cancer cells via nuclear translocation of FOXO3 and ROS level elevation [60]. In view of these findings, these lncRNAs may have a profound effect on the regulation of FAdV-4 infection-induced host cell apoptosis by regulating their target genes. In the validation of the predicted lncRNA−mRNA pairs related to apoptosis, we found that BMP4, a lncRNA 54128 target gene, was upregulated. Subsequently, to study the role that lncRNAs play in the process of FAdV-4-induced apoptosis, lncRNA 54128 was selected for further study from the 71 identified lncRNAs whose functions were predicted to be involved in apoptosis. Our results indicated that lncRNA 54128 interference significantly suppressed the transcription of BMP4, a target gene of lncRNA 54128, and suppressed the rate of apoptosis. These findings provide a better understanding of the potential association between lncRNAs and FAdV-4 infection. As we only verified the regulatory function of lncRNA 54128 through a loss-of-function experiment, the signaling pathways and mRNAs involved in the regulation of FAdV-4 infection-induced apoptosis by lncRNA 54128 need to be further explored.

## 5. Conclusions

In this study, we first obtained the transcription profiles of lncRNAs and related mRNAs in FAdV-4-infected LMH cells based on transcriptome analysis. This study indicated the importance of the lncRNA landscape in virus−host interactions as a total of 1798 DE lncRNAs were identified. Based on bioinformatics tools, we found 71 apoptosis-related lncRNAs. In addition, our experimental data showed the role of lncRNA 54128 in regulating FAdV-4-induced apoptosis. This study provides ideas for future studies of the molecular mechanisms underlying the pathogenesis of FAdV-4 infection.

## Figures and Tables

**Table 1:** Primers used in this study.

Primers	Sequences (5′–3′)
ENSGALG00000054128	GGACACATCTCTTTCTTGCCC
	ACGTGATTAAGGGCAGCAGAT
ENSGALG00000054172	GCAGGATGCCAGTTCAGAGT
	TGCCTTTCTCAGTTATTCCAGT
XLOC_026155	TGCACTTAGTAGCAGTATAGCCA
	TTCAAGGTCCTAGCCTCCCA
ENSGALG00000050472	TGCGGAGTAATAGGTCAGTGAG
	TCCAGCCTTCTGCATTCTCC
ENSGALG00000048532	AAGCATGTGGCAACTTCGTA
	ACTGATCAGCTGCCATTTATGA
BMP4	CGCTCCTGGTCACCTTCG
	CAACCCACGTCGCTGAAATC
SOCS2	GTACCAGGACGGCAAGTTCA
	TAGAGGTGGACCGTCCCATT
FOXO3	CCCATGATGTCGTTTGCTGC
	CCGCTAAGAGGAGAGCTGTG
SLC40A1	CTAGGGTTGGCCTTTGGTCC
	GCCTCTTTCAGATTCCGCCA
GAPDH	GAGGGTAGTGAAGGCTGCTG
	CATCAAAGGTGGAGGAATGG

**Table 2:** Data quality of lncRNA and mRNA profiles and clean reads compared with the reference genome.

Sample	Raw Reads	Clean Reads	Q30 of Clean Reads (%)	Total Mapped Reads (%)
M-1	93,753,712	93,689,766	94.25	92.545
M-2	107,934,056	107,443,430	93.77	91.440
M-3	94,306,864	93,891,362	94.17	90.616
24P-1	108,867,196	108,460,480	92.28	73.424
24P-2	102,718,926	102,619,998	94.07	77.610
24P-3	113,636,874	113,200,420	93.98	73.189

**Table 3:** The top 20 lncRNA–mRNA pairs related to apoptosis.

LncRNA_Name	LncRNA_Name Fold Change	mRNA_Name	Target mRNA Fold Change	Correlation
XLOC_026155	−11.64	FOXO3	−1.86	0.990
ENSGALG00000037194	3.99	RHOB	1.54	0.998
XLOC_003734	8.41	MKP3	2.82	0.986
ENSGALG00000052384	1.59	TNFRSF18	2.19	0.986
XLOC_000612	3.14	MKP3	2.82	0.984
ENSGALG00000037919	3.89	RHOB	1.54	0.983
XLOC_032168	2.57	FOS	2.81	0.979
ENSGALG00000050472	−1.86	FOXO3	−1.86	0.978
XLOC_027548	9.33	ENSGALG00000031518	2.90	0.978
XLOC_033831	2.47	BMP4	3.41	0.977
ENSGALG00000047387	9.09	CRYAA	3.06	0.975
ENSGALG00000049522	4.70	ENSGALG00000031427	2.75	0.975
XLOC_034149	2.50	PAX2	1.90	0.974
ENSGALG00000048532	−2.46	SLC40A1	−1.85	0.969
ENSGALG00000050121	2.38	TNFAIP3	3.89	0.957
XLOC_014249	2.30	HOXA5	2.20	0.949
ENSGALG00000054128	1.95	BMP4	3.41	0.935
ENSGALG00000051113	3.73	FASLG	1.59	0.921
XLOC_035991	1.72	NR4A2	2.26	0.918
XLOC_027850	3.89	ENSGALG00000031518	2.90	0.910

**Table 4:** Validation of the LncRNA–mRNA pairs by qPCR.

LncRNA_id	LncRNA_seq Fold Change	Real-Time PCR Fold Change	mRNA Symbol	mRNA_seq Fold Change	Real-Time PCR Fold Change
ENSGALG00000054128	1.95	3.56	BMP4	3.41	13.7
ENSGALG00000054172	2.66	4.45	SOCS2	1.26	2.82
XLOC_026155	−11.64	−2.39	FOXO3	−1.86	−2.51
ENSGALG00000050472	−1.8	−1.58	FOXO3	−1.86	−2.51
ENSGALG00000048532	−2.46	−3.7	SLC40A1	−1.85	−2.44

## Data Availability

The datasets generated in this study were submitted to the Gene Expression Omnibus (GEO) database with accession number PRJNA729519 (ID: 9560975).

## Supplementary Materials

The following are available online at https://www.mdpi.com/article/10.3390/v13081623/s1, Table S1: Identification of 71 DE lncRNAs related to apoptosis.
